# SNP Discovery from Transcriptome of the Swimbladder of *Takifugu rubripes*

**DOI:** 10.1371/journal.pone.0092502

**Published:** 2014-03-20

**Authors:** Jun Cui, Hongdi Wang, Shikai Liu, Lifu Zhu, Xuemei Qiu, Zhiqiang Jiang, Xiuli Wang, Zhanjiang Liu

**Affiliations:** 1 Key Laboratory of Mariculture & Stock Enhancement in North China's Sea, Ministry of Agriculture, Dalian Ocean University, Dalian, China; 2 The Fish Molecular Genetics and Biotechnology Laboratory, Aquatic Genomics Unit, School of Fisheries, Aquaculture and Aquatic Sciences and Program of Cell and Molecular Biosciences, Auburn University, Auburn, Alabama, United States of America; 3 School of Environmental and Chemical Engineering, Dalian Jiaotong University, Dalian, China; Chinese Academy of Fishery Sciences, China

## Abstract

Single nucleotide polymorphisms (SNPs) have become the marker of choice for genome-wide association studies in many species. High-throughput sequencing of RNA was developed primarily to analyze global gene expression, while it is an efficient way to discover SNPs from the expressed genes. In this study, we conducted transcriptome sequencing of the swimbladder of *Takifugu rubripes* using Illumina HiSeq2000 platform to identify gene-associated SNPs in the swimbladder. A total of 30,312,181 unique-mapped-reads were obtained from 44,736,850 raw reads. A total of 62,270 putative SNPs were discovered, which were located in 11,306 expressed genes and 2,246 scaffolds. The average minor allele frequency (MAF) of the SNPs was 0.26. GO and KEGG pathway analysis were conducted to analyze the genes containing SNPs. Validation of selected SNPs revealed that 54% of SNPs (26/48) were true SNPs. The results suggest that RNA-Seq is an efficient and cost-effective approach to discover gene-associated SNPs. In this study, a large number of SNPs were identified and these data will be useful resources for population genetic study, evolution analysis, resource assessment, genetic linkage analysis and genome-wide association studies.

## Introduction

Next-generation sequencing-based RNA-Seq analyses have dramatically changed the way to investigate the functional complexity of transcriptome in many organisms [1], [2]. RNA-Seq approach is powerful for unraveling transcriptome complexity, identification of genes, gene-associated markers, regulatory non-coding RNAs and for alternative splicing analysis and expression profiling [3]–[5]. Transcriptome analysis using the next generation sequencing technologies have been widely reported in many species, including several aquaculture species such as catfish [6]–[8], Atlantic cod [9], silver carp [10], pearl oyster [11], carp [12], and Amur ide [13].

Recently, RNA-Seq has also been used as an efficient and cost-effective method to comprehensively identify SNPs from transcribed regions in the genomes of several fish species. By sequencing of the pooled RNA samples from multiple individuals of channel catfish and blue catfish, a set of quality SNPs were identified including 342,104 intra-specific SNPs for channel catfish, 366,269 intra-specific SNPs for blue catfish, and 420,727 inter-specific SNPs between channel catfish and blue catfish [6]. Similarly in carp, a total of 712,042 intra-stain SNPs were discovered in four strains, including mirror carp (483,276 SNPs), purse red carp (486, 629SNPs), Xingguo red carp (478,028 SNPs), and Yellow River carp (488,281 SNPs) [14]. Large sets of SNPs have also been reported in some other aquaculture species, such as the Eastern oyster [15], Atlantic salmon [16], Atlantic cod [9] and rainbow trout [17].

*Takifugu rubripes*, widely distributed in the Asia, is one of the most important aquaculture species in China. In our laboratory, some SNPs makers associated with growth traits have been identified from the growth-related genes including *Leptin*, *Melanocortin 4 Receptor* (*MC4R)*, *Insulin-like growth factor* (*IGF*), *Myogenic factor 5* (*Myf5*), *Growth hormone releasing hormone* (*GHRH*), *Myogenic factor 6* (*Myf6*) [18]. Other genetic and genomic studies were also conducted with the focus on identification and characterization of microsatellite markers [19], [20], construction of bacterial artificial chromosome (BAC) and expressed sequence tag (EST) library [21]. In addition to its importance in aquaculture, *T. rubripes* is also widely used as a model system in many scientific fields, especially in the evolutionary studies. The fugu genome has been completed, which is among the smallest vertebrate genomes. It has proven to be a useful ‘reference’ genome for identifying genes and other functional elements in human and other vertebrate genomes, and for understanding the structure and evolution of vertebrate genomes [22]–[24].

The swimbladder in teleost fish is a specialized organ that regulates buoyancy. The homology of the fish swimbladder and mammalian lung has been well recognized based on morphological and embryological evidence. However, the molecular evidence of homology of swimbladder and the mammalian lung was not sufficient [25]–[27]. A large set of SNPs from the swimbladder transcriptome of *T. rubripes* should provide valuable resources for swimbladder research, lung research and evolution studies of fish swimbladder and mammalian lung.

In this study, we sequenced the transcriptome of the swimbladder of *T. rubripes* using Illumina HisSeq2000 platform to identify gene-associated SNPs. A total of 62,270 putative SNPs were discovered, which were located in 11,430 genes and 1,612 scaffolds, and the average minor allele frequency (MAF) was 0.26. These SNPs should provide useful resources for evolution, population genetic study, resource assessment, genetic linkage analysis and genome-wide association studies.

## Results and Discussion

### Transcriptome sequencing

Illumina sequencing was conducted to generate short sequence reads from the swimbladder of *T. rubripes*. A total of 30,312,181 unique-mapped-reads were obtained from 44,736,850 raw reads after being mapped to the fugu *T. rubripes* fifth genome assembly from Ensembl database. The genome distribution of the uniquely mapped reads was assessed based on the RefSeq-defined gene models. As expected, the majority of reads (60%) were mapped onto exonic regions, while a large propotion of reads were mapped onto intergenic regions (Table 1). Similar observations have been reported in the studies of mouse and *Caenorhabditis elegans* [28], [29]. The RNA-Seq data in this study has been deposited in the NCBI SRA database with the accession number of SRR1022677.

**Table 1:** The genome distribution of the mapped reads.

Read distribution	Number of reads	Percentage
Exonic region	18,120,867	59.78%
Intergenic region	9,776,865	32.25%
Intronic region	1,435,258	4.73%
Exon-intron junction	176,167	0.58%

### SNP identification

Compared with the fugu genome, a total of 62,270 putative SNPs were identified. The detailed SNP information was provided in Table S1. Of which, the number of homozygotes was 9,518 and the number of heterozygotes was 52,752. In these heterozygotes, the C/T and A/G were the most common types. In contrast, G/T, A/C, G/C and A/T were the lease common types (Table 2).

**Table 2:** Summary of SNP types identified from the *T. rubirpes* swimbladder.

SNP type	Number
Homozygote	
A	1,887
C	2,914
G	2,887
T	1,830
Heterozygote	
G/T	4,730
A/C	4,725
A/G	16,972
G/C	4,665
A/T	4,586
C/T	17,074
Total	62,270

The SNPs were classified into several categories based on their locations in the genome, including inter-genic, down_stream (+1k), exon, intron, and up_stream (−1 k). As shown in Table 3, of the 62,270 putative SNPs, 24,525 SNPs (39.38%) were identified in exons, which were highly represented, while 4,210 SNPs (6.76%) were identified in the introns, which were lowly represented.

**Table 3:** Classification of putative SNPs.

SNP classification	Number of putative SNPs
Inter-genic	12,903
Down_stream(+1 k)	12,303
Exon	24,525
Intron	4,210
Up_stream(−1 k)	8,329
Total	62,270

Inter-genic SNPs were identified from regions between genes, while Down_stream(+1 k) and Up_stream(−1 k) represents SNPs identified from regions of 1 kb downstream and upstream of the genes.

### Minor allele frequency distribution

**Figure 1:**
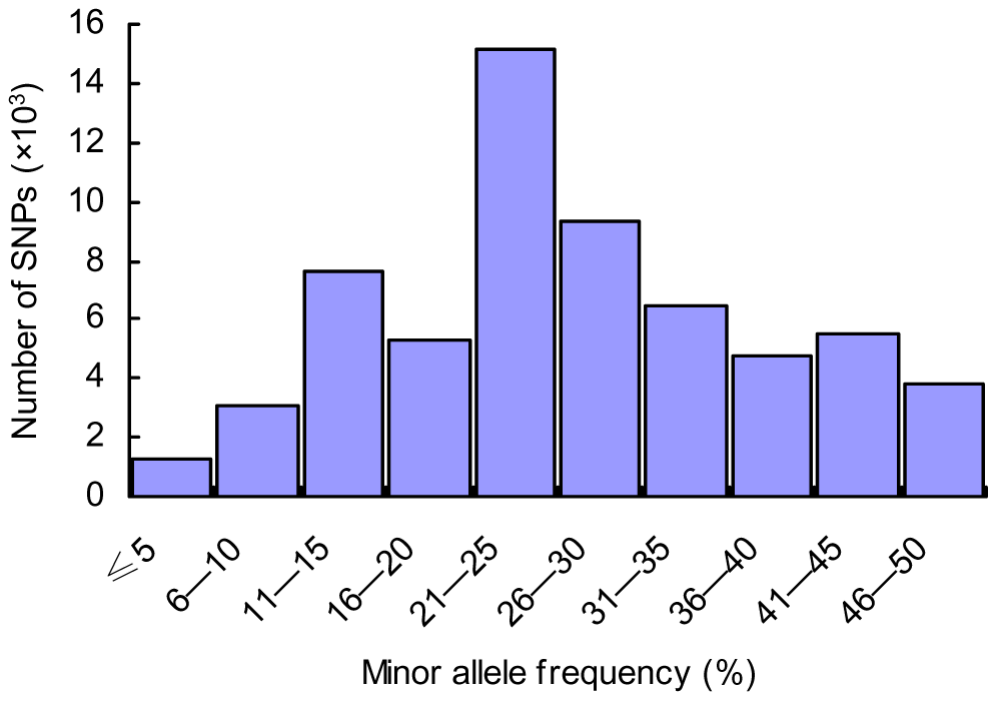
Distribution of minor allele frequencies (MAFs) of SNPs identified from the *T. rubirpes* swimbladder. The X-axis represents the SNP minor allele frequency in percentage, while the Y-axis represents the number of SNPs with given minor allele frequency

Minor allele frequency (MAF) is an important factor for SNP locus evaluation. MAFs of SNPs were calculated from the sequence data. As shown in Figure 1, the majority of SNPs have sequence derived minor allele frequencies ranging from 21% to 25%, and the average MAF was 26% in putative SNPs identified from the swimbladder of *T. rubripes*.

### SNP distribution among genes and scaffolds

**Figure 2:**
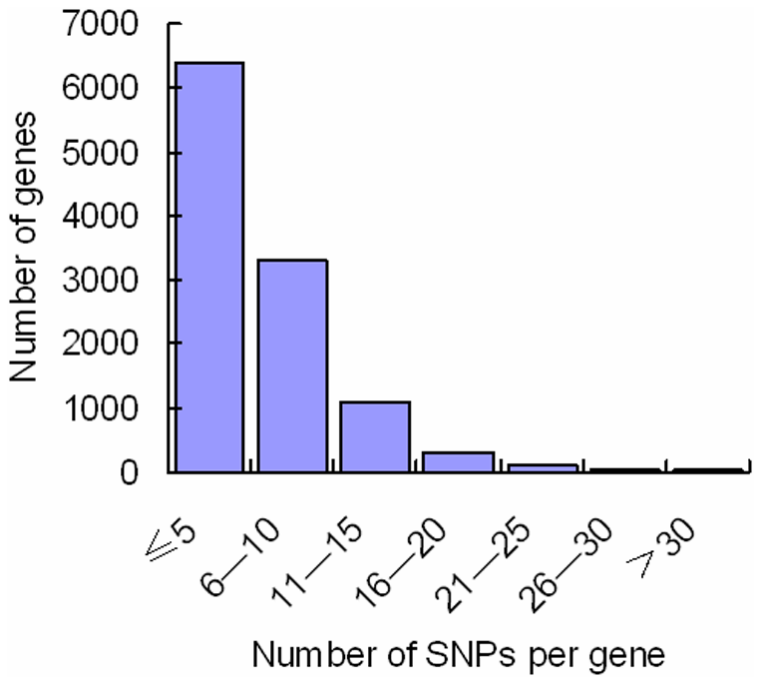
SNP distribution among genes. The X-axis represents gene size (number of SNPs per gene)

**Figure 3:**
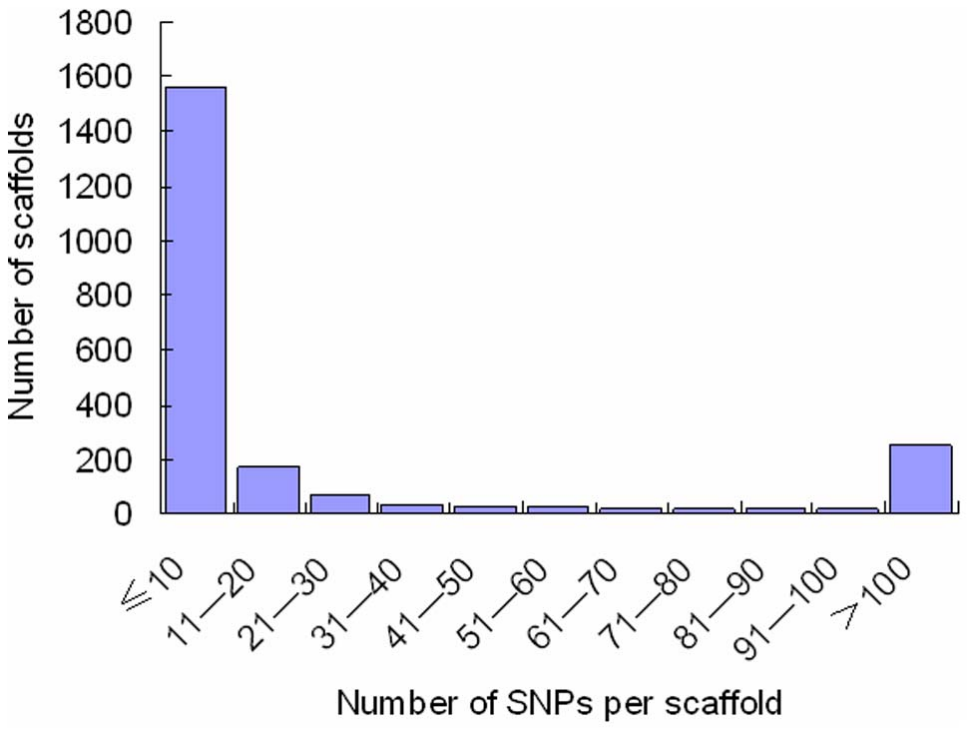
SNP distribution among scaffolds. The X-axis represents scaffold size (number of SNPs per scaffold)

SNPs distribution is important for consideration of coverage when using SNP makers. The distribution of SNPs in the genes was analyzed. Expressed short reads were mapped to a total of 17,249 genes based on the fifth fugu *T. rubripes* genome assembly from Ensembl database. On average, 3.6 SNPs per gene were identified. A total of 11,306 expressed genes containing SNPs were identified in the swimbladder with the cutoff values of PRKM setting as 0.08. As shown in Figure 2, of these genes, 56.73% had fewer than 5 SNPs per gene. The number of genes with 26–30 SNPs per gene is 40 and there are 30 genes harboring more than 30 SNPs per gene. For instance, the dystonin (ENSTRUG00000015507) and annexin A5 (ENSTRUG00000015464) have relatively large numbers of SNPs per gene, 73 and 63 SNPs, respectively. The fugu genome assembly (version 5.0) is composed of 7,119 scaffolds. The SNPs identified in the present study were found on the 2,246 scaffolds, about 27.7 SNPs per scaffold. As shown in Figure 3, a large number of scaffolds had fewer than 10 SNPs per scaffold. The scaffold_1 and scaffold_6 had the largest number of SNPs, 1,631 and 1,293 SNPs, respectively.

### Gene Ontology and KEGG pathway analysis

**Figure 4:**
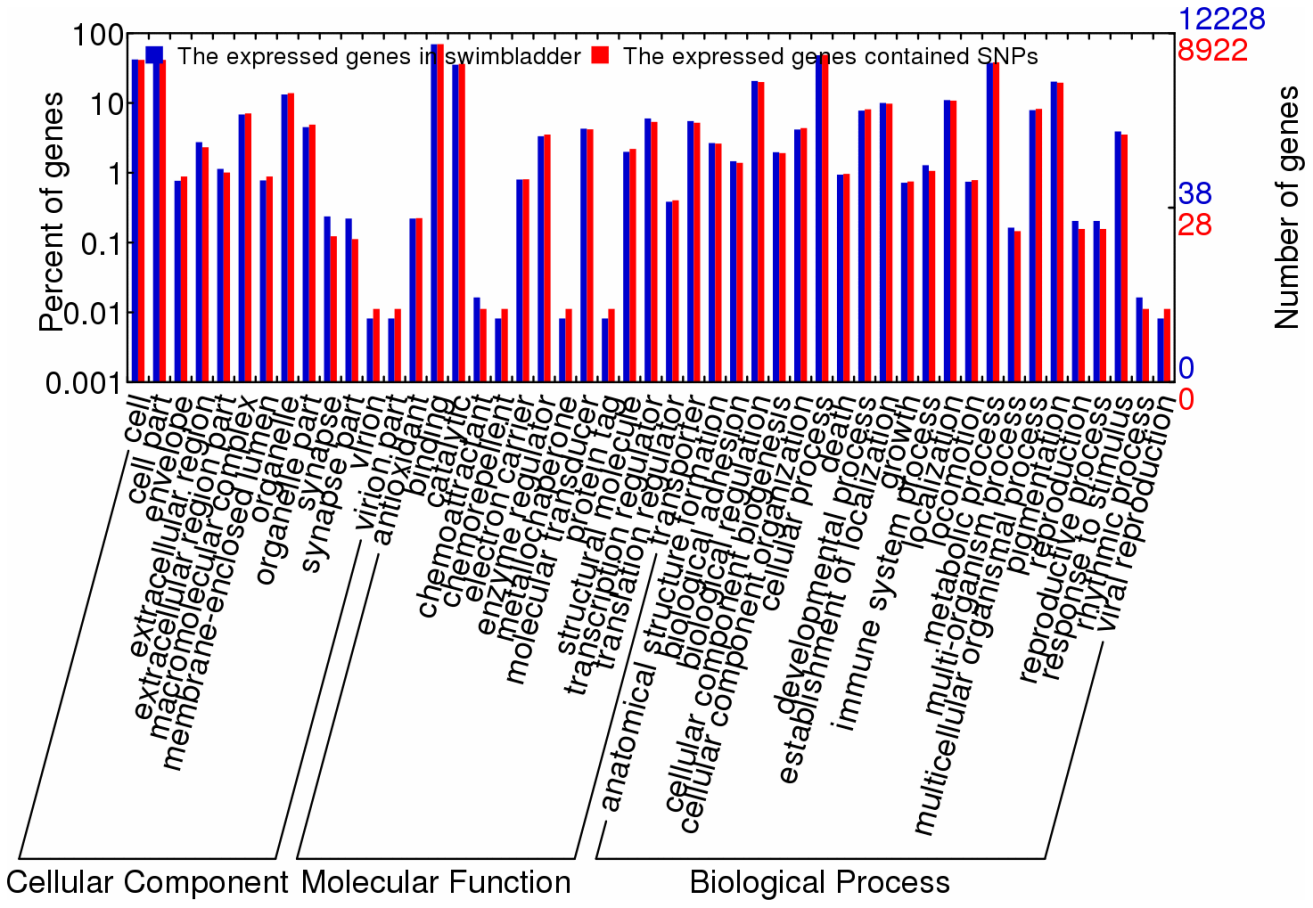
Gene Ontology of genes containing putative SNPs.

Gene Ontology (GO) annotation was further performed for the annotated genes in terms of biological process, molecular function and cellular component. Distribution of the genes in different GO categories at level 2 is shown in Figure 4. In the swimbladder, 8,922 expressed genes containing SNPs were assigned with one or more GO terms for biological process, molecular function and cellular component. For biological process, genes involved in the metabolic process and cellular process were highly represented. For molecular function, binding was the most represented GO term, followed by catalytic activity. Regarding to the cellular component, the major categories were cell and cell part. The GO categories of expressed genes containing SNPs were in the same proportion to the GO categories of all the expressed genes (Figure 4).

Besides GO analysis, KEGG pathway analysis was also carried out for the annotated genes, which is an alternative approach to categorize gene functions with the focus on biochemical pathways. A total of 3,808 expressed genes were assigned with one or more KEGG annotation and were mapped to KEGG pathways (Table 4). Of these annotated genes, 28.06% were classified into the Organismal Systems with the majority of which involved in immune system. Metabolism pathways including carbohydrate metabolism, amino acid metabolism and lipid metabolism represented 25.66%. Environmental information processing represented 19.41%. The signal transduction was one of the well-represented sub-pathways. In addition, 9.15% and 17.72% were classified into the Genetic information processing and Cellular Processes, respectively.

**Table 4:** KEGG biochemical mappings for genes containing SNPs.

KEGG categories	Number of genes
**Metabolism**	
Amino Acid Metabolism	344
Biosynthesis of Polyketides and Nonribosomal Peptides	4
Biosynthesis of Secondary Metabolites	69
Carbohydrate Metabolism	422
Energy Metabolism	162
Glycan Biosynthesis and Metabolism	150
Lipid Metabolism	331
Metabolism of Cofactors and Vitamins	130
Metabolism of Other Amino Acids	103
Nucleotide Metabolism	239
Xenobiotics Biodegradation and Metabolism	118
**Genetic Information Processing**	
Folding, Sorting and Degradation	266
Replication and Repair	161
Transcription	196
Translation	116
**Environmental Information Processing**	
Membrane Transport	39
Signal Transduction	1142
Signaling Molecules and Interaction	386
**Cellular Processes**	
Behavior	28
Cell Communication	522
Cell Growth and Death	365
Cell Motility	200
Transport and Catabolism	316
**Organismal System**	
Circulatory System	138
Development	187
Endocrine System	544
Immune System	1116
Nervous System	246
Sensory System	35

### Homologous genes containing SNPs between fugu swimbladder and human lung

In this study, our KEGG pathway analysis indicated the tight junction existed, including 141 expressed genes containing SNPs. **Tight junction** is essential for epithelial morphology and function of swimbladder. Tight junctions serve to form seals among epithelial cells, creating a selectively permeable barrier to intercellular diffusion [27]. Claudins are transmembrane proteins which act in concert with other transmembrane and peripheral proteins to form the physical basis for tight junction [27], [30]. In previous studies, *claudin 4/5/6/7/9* genes were identified in the swimbladder of zebrafish [27] and 46 claudin genes in the fugu genome were identified and their phylogenetic relationships to those counterparts in mammals was determined [31]. In this study, 16 members of claudin family were identified (Table 5). Three of the 16 **claudin** genes were highly expressed, including claudin 5a, 5b and 7d. In the human airway, *claudin 1*, *3*, *4*, *5* and *7* are expressed in both bronchi and bronchioles. *Claudin 5* is localized exclusively in the apical-most region of the tight junctions. Altered *Claudin* expression pattern can change the paracellular permeability characteristics of the epithelium. *Claudin 5* overexpression increases the solute permeability [32], [33]. Genome wide association studies showed the polymorphisms rs9290927, rs893051 and rs17501010 from *clandin 1* were associated with nickel contact sensitization in individuals without ear piercings, contact sensitization to fragrances, and with both organic compounds and nickel contact dermatitis in human, respectively [34]. The genetic variants in regulatory regions of *clandin 1* can alter susceptibility to HCV infection [35].

**Table 5:** Identification of expressed *Claudin* genes containing SNPs.

Ensembl Gene ID	Gene name	RPKM value	Number of SNPs
ENSTRUG00000018609	*Claudin 5b*	234.65	5
ENSTRUG00000016497	*Claudin 5a*	75.65	5
ENSTRUG00000007521	*Claudin 7a*	43.24	6
ENSTRUG00000010140	*Claudin 30c*	14.33	3
ENSTRUG00000004991	*Claudin 12*	8.98	6
ENSTRUG00000011829	*Claudin 11a*	6.15	4
ENSTRUG00000015308	*Claudin 15a*	3.81	1
ENSTRUG00000003031	*Claudin 25*	2.89	2
ENSTRUG00000010901	*Claudin 23*	1.83	4
ENSTRUG00000001287	*Claudin 19*	1.63	4
ENSTRUG00000013204	*Claudin 18*	1.21	2
ENSTRUG00000007366	*Claudin 32a*	0.78	3
ENSTRUG00000011741	*Claudin 31*	0.76	2
ENSTRUG00000009832	*Claudin 28b*	0.31	1
ENSTRUG00000016459	*Claudin 15b*	0.21	2
ENSTRUG00000010378	*Claudin 23b*	0.14	1

In this study, 8 *Wnt* genes containing SNPs were identified and the expression levels of *wnt 7b*, *wnt 5a* and *wnt 11* are higher (Table 6). Wnt signaling pathway has been reported to play important roles in mammalian lung development [36]–[38]. In previous studies, the down-regulation of Wnt signaling leading to defective swimbladder development in zebrafish was observed [39]. *Wnt7b* is expressed in the distal airway epithelium of lungs and plays critical roles in lung development such as distal epithelial cell fate decision, lung mesenchymal proliferation and smooth muscle differentiation [38], [40]–[43]. It was found that *wnt5a* is expressed in lung epithelium [38], [44]. *Wnt11* plays important roles in mouse lung development [38], [45], [46]. In chicken, 124 SNPs from 31 genes of Wnt signaling pathway were selected to genotype in 764 individuals resulted in 102 polymorphic SNPs [47]. In human, 14 SNPs from six Wnt pathway-related genes were genotyped in 210 individuals (145 men and 65 women), including *Dickkopf 2* (*DKK2*) (rs17037102, rs419558, and rs447372), *DKK3* (rs3206824, rs11022095, rs1472189, rs7396187, and rs2291599), *DKK4* (rs2073664), *sFRP4* (rs1802073 and rs1802074), *SMAD7* (rs12953717), and *DAAM2* (rs6937133 and rs2504106) [48]. Six common SNPs of *Wnt10b* were identified in a sample of 1,029 Korean female subjects, which were in almost complete linkage disequilibrium [49].

**Table 6:** Identification of expressed *Wnt* genes containing SNPs.

Ensembl Gene ID	Gene name	RPKM value	Number of SNPs
ENSTRUG00000016453	*Wnt 7b*	48.59	1
ENSTRUG00000001530	*Wnt 11*	29.03	8
ENSTRUG00000008614	*Wnt 5a*	28.52	7
ENSTRUG00000000172	*Wnt 4*	16.51	4
ENSTRUG00000003640	*Wnt 6*	11.5	14
ENSTRUG00000016522	*Wnt 5b*	1.71	2
ENSTRUG00000014284	*Wnt 9b*	1.38	3
ENSTRUG00000012568	*Wnt 2b*	0.33	1

We observed the expression of two homologues of *Ihh* (ENSTRUG00000012233 and ENSTRUG00000013525) and *Ptc1* (ENSTRUG00000014514) containing SNPs from the swimbladder transcriptome. The role of Hh (Hedgehog) signaling pathway in lung development is very crucial in human, mouse, chicken and *Xenopus laevi*s [38], [50]–[53]. Some development-related genes in lung had been identified in zebrafish, such as Sonic Hedgehog (*Shh*)-related gene, Indian Hedgehog (*Ihh*)-related gene and their receptors, *Patched 1*(*Ptc 1*) and *Ptc2* [54]-[61]. The human sonic hedgehog (*SHH*) gene is located in the 7q36 region, which is known to play an important role in embryo patterning, lung development and connection with sexual orientation. A SNP site (rs9333613) was found to be associated with male sexual orientation [62]. *Ihh* is a good candidate gene for association study of developmental disorders mainly affecting skeleton development. The previous study showed that the SNP sites of *Ihh* were associated with equine bone developmental disorders [63].

### SNP validation

As the SNPs reported in the present study were identified through bioinformatic analysis, the results were needed to evaluate for the validation rate. A total of 48 SNPs were randomly selected for validation by PCR amplification and Sanger sequencing [64]. Of the 48 SNPs, 26 SNPs (54%) were validated and 22 SNPs were not found by PCR amplification and direct sequencing (Table 7).

**Table 7:** Summary of SNP validation.

Ensembl Gene ID	Gene Name	Number of SNPs tested	Number of SNPs validated
ENSTRUG00000011255	Translocase of outer mitochondrial membrane 20 homolog	5	2
ENSTRUG00000008698	RAB9A, member RAS oncogene family	7	4
ENSTRUG00000014751	Family with sequence similarity 46, member A	5	3
ENSTRUG00000009192	Coiled-coil domain containing 47	5	2
ENSTRUG00000006299	Mitochondrial ribosomal protein L21	8	5
ENSTRUG00000006704	Calpain small subunit 1	5	3
ENSTRUG00000004026	C-type lectin domain family 11, member A	6	3
ENSTRUG00000014304	Proliferating cell nuclear antigen	7	4

## Materials and Methods

### Ethics statement

This study was approved by the Animal Care and Use committee of Key Laboratory of Mariculture & Stock Enhancement in North China's Sea at Dalian Ocean University. All surgery was performed under sodium pentobarbital anesthesia, and all efforts were made to minimize suffering.

### Sample collection and RNA isolation

A total of 45 *Takifugu rubripes* (length 20cm) were sampled from Dalian Tianzheng Industrial Co., Ltd (Dalian China). The swimbladders of these fish were collected and pooled. Tissues were placed into RNAlater (Ambion), stored at room temperature for 24 h, and then moved to −80°C for storage until RNA isolation. Total RNA was extracted from the pooled swimbladder using the TRIzol R Reagent (Invitrogen, CA, USA) by following the manufacturer's protocol. The quantity and quality of total RNA was measured using an Agilent 2100 Bioanalyzer.

### cDNA library construction and sequencing

Total RNA was sent out for next generation sequencing provided by Beijing Institute of Genomics, Chinese Academy of Sciences. cDNA libraries were constructed from mRNA from swimbladder. cDNA libraries were prepared using the Illumina TruSeq RNA Sample Preparetion Kit (Illumina) according to the TruSeq protocol. After KAPA quantitation and dilution, the libraries were clustered 3 per lane and sequenced on an Illumina HiSeq 2000 instrument with 100 bp paired-end reads.

### Reads mapping

The reads were mapping to the fugu *T. rubripes* fifth genome assembly by BWA program. During the mapping phase, up to five mismatches were allowed. The expression levels (RPKM, Reads Per Kilobase of exon model per Million mapped reads) for each gene were calculated using uniquely mapped reads by in-house Perl script according to the equation:
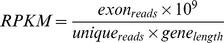



The cutoff value of gene expression was calculated for each sequencing sample, genes with RPKM greater than cutoff value were defined as expressed genes [65].

### SNP identification

BWA and SAMtools (Tools for alignments in the SAM format) software were used to align reads to the fugu genome assembly (version 5.0) for detecting SNPs [66], [67]. Filtering thresholds were set as: consensus quality is no less than 20 and coverage is no less than 10.

### Gene Ontology and KEGG pathway analysis

Gene Ontology (GO) and KEGG pathway analyses were conducted to the genes containing SNPs. GO annotation analysis was performed using Blast2GO, an automated tool for the assignment of GO terms. The annotation result was categorized with respect to Biological Process, Molecular Function, and Cellular Component at level 2. In order to gain an overview of gene pathway networks, KEGG analysis was performed using the online KEGG Automatic Annotation Server (KAAS) (http://www.genome.jp/kegg/kass/). The bi-directional best hit (BBH) method was used to obtain KEGG orthology assignments.

### SNP validation

To evaluate the validation rate of the SNPs identified by bioinformatic analysis, we randomly selected 48 SNPs and validated by PCR amplification and direct sequencing. PCR primers were designed according to the assembled transcript sequences and were listed in the Table S2. Ten individuals were used for the SNP validation.

## Conclusions

In this study, a large number of SNPs were identified by the transcriptome sequencing of the *T. rubirpes* swimbladder using Illumina HiSeq2000 platform. A large proportion of randomly selected SNPs were verified using the Sanger sequencing, suggesting the high validation rate. The SNPs should provide valuable resources for genomic studies, evolution analysis, population genetic study, resource assessment, genetic linkage analysis and genome-wide association studies.

## Supporting Information

Table S1 **The SNPs identified from the transcriptome of the swimbladder of** ***Takifugu rubripes*** **.**
(TXT)

Table S2 **Primers used for SNP validation in the study.**
(DOC)
