# Supplementary material for: SNP Discovery from Transcriptome of the Swimbladder of Takifugu rubripes
Source: PLoS One. 2014 Mar 20;9(3):e92502. doi: 10.1371/journal.pone.0092502 (PMC3961390; doi:10.1371/journal.pone.0092502)
Supplement: Table S2 — Primers used for SNP validation in the study. (DOC) [file pone.0092502.s002.doc]

**Table S2:** Primers used for SNP validation in the study

| **Gene ID** | **Gene name** | **Primer sequences (5'-3)'** | **Tm (℃)** |
| --- | --- | --- | --- |
| ENSTRUG00000011255 | translocase of outer mitochondrial membrane 20 homolog | F: AGGAGCAGTGAGACGGGAGC  R: ACGCCATGATGGGAGTGA | 55 |
| ENSTRUG00000008698 | RAB9A, member RAS oncogene family | F: CAAATGATCCGCTCACCA  R: TCGCTGGAATTGAATTGTCT | 60 |
| ENSTRUG00000014751 | family with sequence similarity 46, member A | F: CAGTTACAGGGCAACCAG  R: AGTTTCGTCCTTGGCATC | 60 |
| ENSTRUG00000009192 | coiled-coil domain containing 47 | F: TCCCATCGTACCTTGAGC  R: TGTCCCGTTGCTTGTCTC | 55 |
| ENSTRUG00000006299 | mitochondrial ribosomal protein L21 | F: AGCGCCTGGAGACAATCT  R: CTCAGGGAGCCAAGGTCA | 58 |
| ENSTRUG00000006704 | Calpain small subunit 1 | F: CCCTCCGAACCTGTGAGT  R: TGCAAAGAACGAGCCAGA | 60 |
| ENSTRUG00000004026 | C-type lectin domain family 11, member A | F: TCCTGCCCTCAGACCTCCCT  R: CCAACCGTGGCGTCCTAA | 58 |
| ENSTRUG00000014304 | proliferating cell nuclear antigen | F: CAACCGCCTGCACTTCTC  F: TGACATCGCAAGCGAGAC | 58 |
